# Length polymorphisms at two candidate genes explain variation of migratory behaviors in blackpoll warblers (*Setophaga striata*)

**DOI:** 10.1002/ece3.5436

**Published:** 2019-07-22

**Authors:** Joel Ralston, Lydia Lorenc, Melissa Montes, William V. DeLuca, Jeremy J. Kirchman, Bradley K. Woodworth, Stuart A. Mackenzie, Amy Newman, Hilary A. Cooke, Nikole E. Freeman, Alex O. Sutton, Lila Tauzer, D. Ryan Norris

**Affiliations:** ^1^ Department of Biology Saint Mary's College Notre Dame IN USA; ^2^ Department of Environmental Conservation University of Massachusetts Amherst MA USA; ^3^ New York State Museum Albany NY USA; ^4^ Department of Integrative Biology University of Guelph Guelph ON Canada; ^5^ School of Biological Sciences The University of Queensland Brisbane Queensland Australia; ^6^ Bird Studies Canada Port Rowan ON Canada; ^7^ Wildlife Conservation Society Canada Whitehorse YT Canada

**Keywords:** *Adcyap1*, avian migration, *Clock*, phenology, pituitary adenylate cyclase‐activating polypeptide

## Abstract

Migratory behaviors such as the timing and duration of migration are genetically inherited and can be under strong natural selection, yet we still know very little about the specific genes or molecular pathways that control these behaviors. Studies in candidate genes *Clock* and *Adcyap1* have revealed that both of these loci can be significantly correlated with migratory behaviors in birds, though observed relationships appear to vary across species. We investigated geographic genetic structure of *Clock* and *Adcyap1* in four populations of blackpoll warblers (*Setophaga striata*), a Neotropical–Nearctic migrant that exhibits geographic variation in migratory timing and duration across its boreal breeding distribution. Further, we used data on migratory timing and duration, obtained from light‐level geolocator trackers to investigate candidate genotype–phenotype relationships at the individual level. While we found no geographic structure in either candidate gene, we did find evidence that candidate gene lengths are correlated with five of the six migratory traits. Maximum *Clock* allele length was significantly and negatively associated with spring arrival date. Minimum *Adcyap1* allele length was significantly and negatively associated with spring departure date and positively associated with fall arrival date at the wintering grounds. Additionally, we found a significant interaction between *Clock* and *Adcyap1* allele lengths on both spring and fall migratory duration. *Adcyap1* heterozygotes also had significantly shorter migration duration in both spring and fall compared to homozygotes. Our results support the growing body of evidence that *Clock* and *Adcyap1* allele lengths are correlated with migratory behaviors in birds.

## 1 INTRODUCTION

Avian migration involves a suite of complex behavioral and physiological responses to exogenous seasonal cues including changes in diet and metabolism, the onset of migratory restlessness (*zugunruhe*), and precise directional orientation (Bairlein, Eikenaar, & Schmaljohann, 2015; Berthold, 1996; Gwinner, 1990; Jenni & Schaub, 2003). The culmination of these responses allow individual birds to accomplish amazing migratory feats, sometimes flying over ocean for several days at a time or navigating thousands of kilometers through unfamiliar habitat between wintering and breeding grounds (Bairlein et al., 2012; Battley et al., 2012; DeLuca et al., 2019, 2015; Gill et al., 2009; McKinnon, Artuso, & Love, 2017). Each of these behavioral and physiological responses is likely genetically inherited (Berthold & Pulido, 1994; Helbig, 1991; Liedvogel & Lundberg, 2014; Pulido, Berthold, Mohr, & Querner, 2001) and could be under natural selection (Gienapp, Leimu, & Merilä, 2007; Nilsson, Klaassen, & Alerstam, 2013; Pulido & Berthold, 2004, 2010). Yet we still know very little about the specific genes or molecular pathways that control these behaviors (Liedvogel, Åkesson, & Bensch, 2011). Further, it is unknown whether molecular pathways controlling migration vary among animal taxa, or even among migratory bird taxa given that migration is evolutionarily labile and likely arose independently multiple times within birds (Pulido, 2007; Zink, 2011). Investigating the genetic control of migratory behaviors in a diversity of bird taxa will therefore allow us to better understand the evolution of migration within birds, as well as ongoing natural selection on these behaviors in response to environmental change (Pulido & Berthold, 2010).

Recent studies have attempted to identify candidate genes that may be linked to migration in insects, birds, and other vertebrates (Contina, Bridge, & Kelly, 2016; Delmore et al., 2015; Johnston, Paxton, Moore, Wayne, & Smith, 2016; Lemopoulos, Uusi‐Heikkilä, Huusko, Vasemägi, & Vainikka, 2018; Lundberg et al., 2013, 2017; Merlin & Liedvogel, 2019; Mueller, Pulido, & Kempenaers, 2011; Steinmeyer, Mueller, & Kempenaers, 2009; Zhu, Gegear, Casselman, Kanginakudru, & Reppert, 2009). Two genes that have received considerable attention, especially in regard to migratory phenology, are *Clock* and *Adcyap1*. *Clock* plays a central role in regulating the circadian oscillator gene complex, which controls circadian and circannual rhythmicity in response to exogenous cues (Panda, Hogenesch, & Kay, 2002; Yu & Hardin, 2006). *Adcyap1* codes for pituitary adenylate cyclase‐activating polypeptide (PACAP) which influences circadian rhythms in part by directly activating *Clock* and other genes in the circadian oscillator complex (Nagy & Csernus, 2007). PACAP also has multiple effects on physiology and behavior throughout the body (Vaudry et al., 2009), including melatonin production in the pineal gland (Csernus et al., 2004) and a role in processing light signals from the retina into neuronal signals (Hannibal et al., 1997), both of which likely play a role in the photoperiodic control of seasonality in birds (Dawson, King, Bentley, & Ball, 2001). *Clock* and *Adcyap1,* therefore, represent two candidate genes that natural selection may act on independently or in concert to shape migratory behaviors in natural populations of migratory organisms.

Several studies have shown a correlation between length polymorphisms in *Clock* and *Adcyap1* and migratory traits in birds (Table 1). For *Clock*, individuals with a greater number of glutamine repeats in the 3′ polyglutamine tail (i.e., longer alleles) exhibit delayed migratory and breeding phenology (Bazzi et al., 2015; Bourret & Garant, 2015; Caprioli et al., 2012; Liedvogel, Szulkin, & Knowles, 2009; Saino et al., 2015) and longer migratory distance (Peterson et al., 2013), relative to individuals with shorter alleles. For *Adcyap1*, longer alleles have been shown to be associated with greater migratory restlessness (Mueller et al., 2011; Peterson et al., 2013), earlier spring arrival dates (Mettler, Segelbacher, & Schaefer, 2015), and earlier postnatal dispersal (Chakarov, Jonker, Boerner, Hoffman, & Krüger, 2013). Additionally, in one species, more northerly breeding populations had longer *Adcyap1* alleles on average than southerly populations (Bazzi et al., 2016), which may reflect geographic variation in migratory strategies or phenological schedules resulting from local adaptation to environmental cues (Johnsen et al., 2007). However, the relationships between candidate genes and migratory phenotypes may also be influenced by local environmental factors such as temperature, photoperiod, and breeding density (Bourret & Garant, 2015), potentially complicating interpretations of geographic patterns within species. Further complicating the study of candidate genes and migratory traits, an increasing number of studies in birds have found no correlation between *Clock* or *Adcyap1* allele length and migratory behavior (Table 1; Bazzi et al., 2016, 2017; Contina, Bridge, Ross, Shipley, & Kelly, 2018; Dor et al., 2012). The interspecific variation in genotype–phenotype correlations for these candidate migratory‐phenology genes highlights the challenge thus far of generalizing the expected relationship between length polymorphism and migration timing, as well as identifying the mechanism by which length variants affect migratory behaviors.

**Table 1:** Studies of association between *Clock* and *Adcyap1* allele lengths, migratory behaviors, and breeding latitude in birds

Gene	Study	Species	Phenology	Migratory propensity	breeding latitude
*Clock*	Johnsen et al. (2007)	Bluethroat, *Luscinia svecica*			0
		Blue tit, *Cyanistes caeruleus*			+
	Liedvogel et al. (2009)	Blue tit, *Cyanistes caeruleus*	+/0^a^		
	Liedvogel and Sheldon (2010)	Great tit, *Parus major*	0		
	Dor et al. (2011)	Barn swallow, *Hirundo rustica*			0
	Mueller et al. (2011)	Eurasian blackcaps, *Sylvia atricapilla*		0	
	Caprioli et al. (2012)	Barn swallow, *Hirundo rustica*	+/0^a^		
	Dor et al. (2012)	Swallows, *Tachycineta*	0		0
	Chakarov et al. (2013)	Common buzzard, *Buteo buteo*	0		
	Peterson et al. (2013)	Dark‐eyed junco, *Junco hyemalis*		+	
	Kuhn et al. (2013)	Pied flycatcher, *Ficedula hypoleuca*	0		0
	Bazzi et al. (2015)	Barn swallow, *Hirundo rustica*	+		
	Bourret and Garant (2015)	Tree swallow, *Tachycineta bicolor*	+		
	Saino et al. (2015)	Nightingale, *Luscinia megarhynchos*	+		
		Pied flycatcher, *Ficedula hypolecua*	0		
		Tree pipit, *Anthus trivialis*	+/0^a^		
		Winchat, *Saxicola ruberta*	0		
	Bazzi et al. (2016)	WILSON'S warbler, *Cardellina pusilla*	0		0
	Bazzi et al. (2017)	Willow warbler, *Phylloscopus trochilus*	0		
	Contina et al. (2018)	Painted bunting, *Passerina ciris*	0	0	
	Romano et al. (2018)	Yellow‐legged gull, *Larus michahellis*	0		
*Adcyap1*	Mueller et al. (2011)	Eurasian blackcaps, *Sylvia atricapilla*		+	
	Chakarov et al. (2013)	Common buzzard, *Buteo buteo*	−		
	Peterson et al. (2013)	Dark‐eyed junco, *Junco hyemalis*		+	
	Bourret and Garant (2015)	Tree swallow, *Tachycineta bicolor*	−/+^b^		
	Mettler et al. (2015)	Eurasian blackcaps, *Sylvia atricapilla*	−/0^a^		
	Saino et al. (2015)	Nightingale, *Luscinia megarhynchos*	0		
		Pied flycatcher, *Ficedula hypolecua*	0		
		Tree pipit, *Anthus trivialis*	0		
		Winchat, *Saxicola ruberta*	0		
	Bazzi et al. (2016)	Wilson's warbler, *Cardellina pusilla*	0		0/+
	Bazzi et al. (2017)	willow warbler, *Phylloscopus trochilus*	0		
	Contina et al. (2018)	Painted bunting, *Passerina ciris*	0	0	
	Romano et al. (2018)	Yellow‐legged gull, *Larus michahellis*	0		

Note

Migratory propensity includes studies of migratory restlessness, distance, and duration. Symbols indicate positive (+), negative (−), or no observed relationships (0).

a Relationships that varied by sex (F/M).

b Relationships that varied by latitude (low latitude/high latitude).

**Figure 1:**
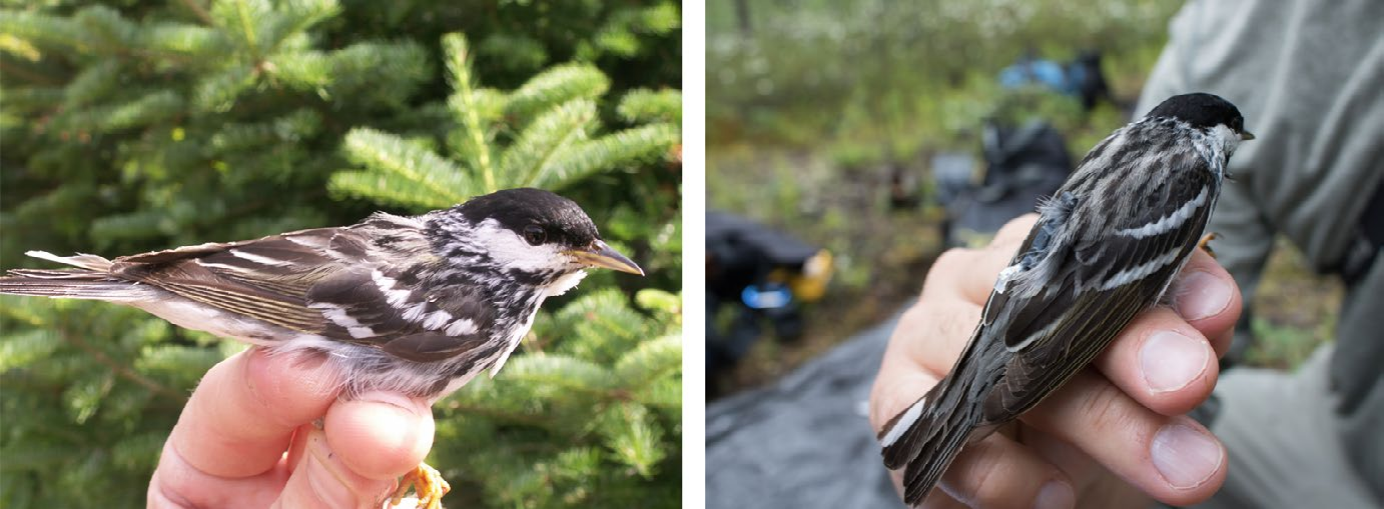
Blackpoll warblers breed across the North American boreal forest and winter in tropical South America. Left: adult breeding male captured in 2010 in New Brunswick. Photo credit: J Ralston. Right: adult breeding male captured in Yukon Territories in 2018, and affixed with a light‐level geolocator. Photo credit: H A Cooke

Given the potential evolutionary and ecological importance of these migratory candidate genes, and the variation in observed patterns across species, it is valuable to continue to build evidence to either support or refute a role of these genes in migratory behavior in different avian species, particularly to help understand why patterns vary across species. Here, we contribute to this growing number of studies by examining relationship between *Adcyap1* and *Clock* and migratory behavior in the blackpoll warbler (*Setophaga striata*), a small (12 g) long distance Neotropical–Nearctic migrant (Figure 1) with geographic variation in migratory behaviors (DeLuca, Holberton, Hunt, & Eliason, 2013; DeLuca et al., 2015; Morris, Covino, Jacobs, & Taylor, 2016).

**Figure 2:**
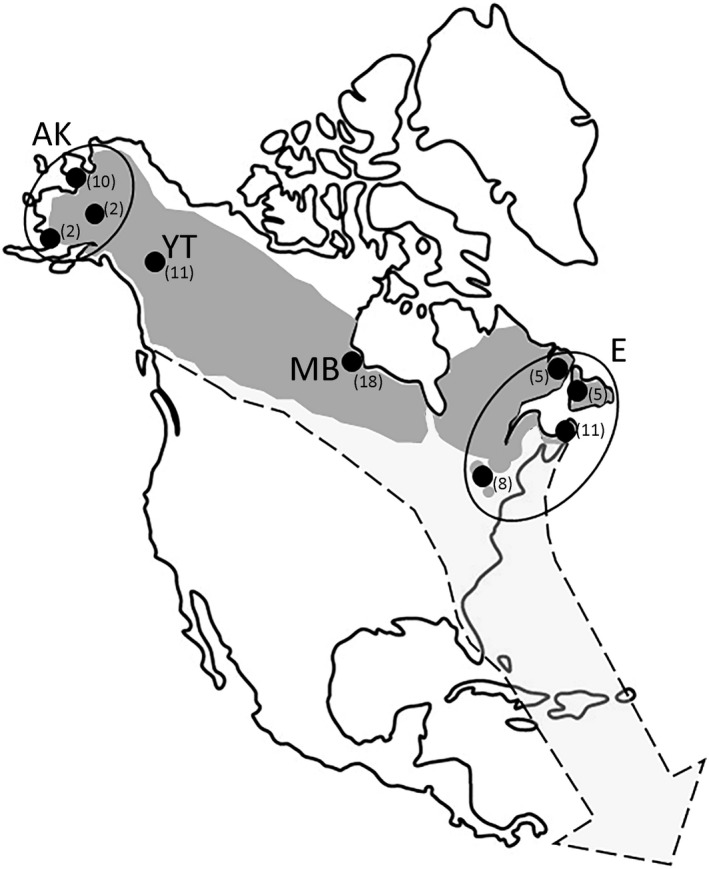
Breeding distribution (dark gray) and fall migratory route (light gray with dashed outline) for blackpoll warblers following DeLuca et al. (2013, 2019, 2015). Sample location is shown with black circles with sample sizes in parentheses. AK, Alaska; YT, Whitehorse, Yukon Territories; MB, Churchill, Manitoba; E, Eastern population. Three sampling sites were grouped together into the AK population: Nome, Denali National Park, and southwest Alaska. Four sampling populations were grouped together into the E population: Adirondack and Catskill Mountains, New York; Cape Breton, Nova Scotia; western Newfoundland; southeastern Labrador

Blackpoll warblers breed across the North American boreal forest from Alaska to Newfoundland and in isolated montane fir forests at the southern periphery of their range in northeastern United States (Figure 2; DeLuca et al., 2013). Annual blackpoll warbler migration between the northern breeding grounds and wintering grounds in the Amazon basin of northern South America is one of the longest migratory routes for any North American songbird (DeLuca et al., 2013; Morris et al., 2016), and was recently tracked from geographically distant breeding populations using light‐level geolocators (DeLuca et al., 2015, 2019; Figure 1). Birds depart the wintering grounds in mid‐April to mid‐May and travel through the Greater Antilles and continental United States to their northern breeding territories, a trip that can vary in duration considerably depending on breeding location (mean 34 days, range 17–49 days; DeLuca et al., 2019). After departing the breeding grounds in August through October, blackpoll warblers first migrate southeastward to the Atlantic coast of United States and Canada, then migrate south over the Atlantic Ocean to their South American wintering grounds, a nonstop trans‐oceanic flight of up to 3,000 km that may take up to three days (DeLuca et al., 2015, 2019).

The large breeding range means that blackpoll warblers from different breeding localities vary in the timing, direction, distance, and duration of their migrations. Birds that breed further north and west migrate further and take longer; these populations tend to depart the wintering grounds earlier but arrive on the breeding grounds at similar times compared to birds breeding further east (DeLuca et al., 2015, 2019). Like in the spring, fall migration timing and duration varies across breeding populations with western breeding birds departing earlier and taking longer to arrive at wintering grounds compared to eastern breeding birds (DeLuca et al., 2015, 2019).

We assessed variation in *Clock* and *Adcyap1* across four breeding populations of blackpoll warbler and tested for correlations between allele lengths and the timing of departure, arrival, and duration in both spring and fall migratory periods. Based on published evidence from other species (Table 1), we predicted allele lengths in both *Clock* and *Adcyap1* would be positively associated with migratory distance and duration. We predicted *Clock* would have a positive relationship with the timing of migration (departure and arrival dates), and *Adcyap1* would have a negative relationship with migratory timing.

## 2 MATERIALS AND METHODS

### 2.1 Genetic sampling

We collected Blackpoll Warbler blood and tissue samples from throughout the breeding range using mist nets during the May–July breeding seasons from 2009 to 2019 (*n* = 64). Blood samples taken in the field were collected using brachial venipuncture and were stored in lysis buffer at ambient temperature until they were transported to a genetics laboratory where they were stored at −20°C. Samples collected in the field were supplemented with a small number of frozen tissues from natural history museum collections, also collected from the breeding seasons from 2008 to 2009 (*n* = 8, Appendix 1). Our total sample (*n* = 72) included only breeding adults, no nestlings, or known relatives, and was mostly male (64/72, 89% males). We extracted DNA from all samples using a DNeasy Blood and Tissue Extraction Kit (Qiagen, Valencia, CA) following the manufacturer's protocol with one modification: Final elution was conducted in two rounds of 75 μl. All blood samples and DNA extracts used in the current study, including those for geolocator tagged birds, are archived at Saint Mary's College, Notre Dame, Indiana, USA, or at New York State Museum, Albany, New York, USA.

We amplified *Clock* and *Adcyap1* loci in 10 μl polymerase chain reactions (PCRs) using a Qiagen Multiplex PCR Master Mix (HotStar Taq DNA Polymerase, dNTP mix, final concentration of 3.0 mM MgCl_2_), 0.2 μM reverse and fluorescent‐labeled forward primers (Applied Biosystems, Foster City, CA), and <200 ng of template DNA. Primer sequences were obtained from Johnsen et al. (2007) and Steinmeyer et al. (2009). Our PCR thermal regime included a 15‐min initial denaturation at 95°C; 35 cycles of 30‐s denaturation at 94°C, 90‐s primer annealing at 54°C, and 90‐s extension at 72°C; and a final extension period of 10 min at 72°C. Final PCR products were mixed with LIZ500 size standard (Thermo Fisher Scientific), diluted in Hi‐Di formamide, and sent to the University of Notre Dame Genomics and Bioinformatics Core Facility for genotyping on an Applied Biosystems 3730xl Genetic Analyzer. We used Peak Scanner (Applied Biosystems) to examine electropherograms and estimate the sizes of the alleles (measurement unit = number of base pairs) from each individual at *Clock* and *Adcyap1* loci.

### 2.2 Analysis of geographic variation

We grouped samples into four populations for analysis: Alaska (*n* = 14), Yukon (*n* = 11), Manitoba (*n* = 18), and Eastern (*n* = 29). The Alaska population included samples from Nome (*n* = 10), Denali National Park (*n* = 2), and southwest Alaska (*n* = 2). Four sampling locations were grouped together into the Eastern population: Adirondack and Catskill Mountains, New York (*n* = 8); Cape Breton, Nova Scotia (*n* = 11); western Newfoundland (*n* = 5); and southeastern Labrador (*n* = 5). All samples from Yukon are from Whitehorse, Yukon Territories, and all samples from Manitoba are from Churchill, Manitoba. Previous analysis of neutral loci revealed significant genetic structure in mitochondrial DNA marker between Newfoundland and other Eastern subpopulations (Ralston & Kirchman, 2012), but no significant structure and a large number of shared alleles at neutral microsatellite markers (Ralston & Kirchman, 2013). Due to the small sample size of Newfoundland in the current study, we group these samples within the Eastern population. We used the package hierfstat (Goudet, 2004) in program R version 3.5.5 (R Core Team, 2019) to calculate overall *F*_ST_ and *F*_IS_. We used the program ARLEQUIN version 3.5 (Excoffier & Lischer, 2010) to test for deviation from Hardy–Weinberg equilibrium, evidence of genetic linkage disequilibrium between the two loci, and to assess genetic population differentiation using pairwise *F*_ST_ and an analysis of molecular variance (AMOVA). Significance for each test was determined using default ARLEQUIN settings for number of permutations and number of steps in Markov chain Monte Carlo (MCMC) algorithms.

We further investigated geographic structure at these loci using the program STRUCTURE version 2.3.4 (Pritchard, Stephens, & Donnelly, 2000), which uses a Bayesian clustering analysis to determine the most likely number of populations (*K*). We used an admixture model with an assumption of correlated allele frequencies among populations, and population of each sample as a prior. We ran 10 iterations for each *K* ranging from 1 to 4 with 1,000,000 MCMC steps following a burn‐in of 100,000 steps, and used mean natural log probability to determine the most likely number of populations. We used the program DISTRUCT (Rosenberg, 2004) to visualize population assignment of each individual from the STRUCTURE run with the greatest log probability for each value of *K*. Additionally, we performed a principal components analysis (PCA) implemented in the R package ade4 (Dray & Dufour, 2007). We visualized clustering of genotypes in ordination space using principal components with eigenvalues greater than 1.0.

While the above genetic analyses assess variation in the frequencies of alleles across populations, they do not directly assess patterns in allele length per se. In other words, in the above analyses, alleles are treated as categorical, while we are also interested in allele length as a continuous variable across geography. We therefore also assessed variation in allele lengths across populations, as well as across latitude and longitude. For these population‐level analyses, we consider latitude and longitude as proxies of migratory behaviors, specifically migratory distance, with the understanding that populations that breed further north and west migrate further each year. We used a series of general linear models (GLMs) with longitude and latitude as the predictor variables to determine whether allele length varied across either sampling latitude or longitude. It is unknown whether there are any allelic interactions or dominance effects at these loci relative to the migratory traits of interest, though previous studies have suggested dominance of the longer allele in *Clock* (Saino et al., 2015) and other genes with 3′ polyglutamine repeats (Ross, 2002). Due to small sample sizes per genotype, and high variability in *Adcyap1*, we could not directly compare genotypes for dominance effects (as in Liedvogel et al., 2009; Saino et al., 2015). However, we did run separate GLMs using either minimum allele, maximum allele, or mean allele length for each individual as the dependent variable. If longer *Clock* alleles are dominant, we would expect to see stronger relationships between individuals' maximum allele length and migratory traits.

### 2.3 Analysis of candidate genes relative to individual migratory traits

To investigate correlations between candidate genes and migratory behaviors at the individual level, we combined genotype data described above with migratory data obtained from light‐level geolocators. In a separate study specifically addressing migratory routes and phenology, DeLuca et al. (2019) tracked migration in blackpoll warblers breeding in Alaska, Yukon, and Manitoba with Biotrack model MK‐6 light‐level geolocators (Wareham, UK), and took blood samples from tagged individuals for the current study (Alaska *n* = 5, Yukon *n* = 4, Manitoba *n* = 8). All individuals used in this analysis were unrelated breeding adult males. Full details on the methods of geolocator deployment and the analysis of geolocator track data are available in DeLuca et al. (2019). For the current study, we analyze six migratory traits related to phenology and duration of migration: (a) spring departure date from wintering grounds, (b) duration of spring migration, (c) spring arrival date on the breeding grounds, (d) fall departure date from breeding grounds, (e) duration of fall migration, and (f) fall arrival date on the wintering grounds.

We tested for correlations between *Clock* and *Adcyap1* allele lengths and each of the six migratory traits using GLMs. To account for known variation in migratory traits across populations (DeLuca et al., 2019), we include population, coded as a factor, as a predictor variable in the models. Like in the population‐level analyses, we built separate GLMs using either minimum allele, maximum allele, or mean allele length for each individual. For six individuals, geolocator track information was not available from the spring migration period. Therefore, analyses comparing spring departure date, spring duration, or spring arrival date with allele length had lower sample sizes (*n* = 12, *n* = 12, *n* = 11, respectively; *n* = 17 for all fall migratory traits). We repeated these analyses using longitude and latitude as covariables instead of population to determine whether our results were robust to this selection.

Next, we tested for an interaction between *Clock* and *Adcyap1* on each of the six migratory traits using GLMs with population, *Clock* allele length, *Adcyap1* allele length, and a *Clock* × *Adcyap1* interaction term as the independent variables. So as to not vastly increase our number of tests by comparing all possible allele length combinations of *Clock* and *Adcyap1*, we only tested GLMs with interactions between allele lengths that showed significant or marginally significant correlations in the individual gene models described above (see Section 3). If we found a significant *Clock* × *Adcyap1* interaction effect on a migratory trait, we do not further discuss single gene effects for this migratory trait.

Lastly, we compared each of the six migratory traits between homozygous and heterozygous individuals for both *Clock* and *Adcyap1* using GLMs with location and heterozygosity as the predictor variables. If heterozygosity correlates with individual fitness and migratory performance (Mettler et al., 2015), we would expect heterozygotes to have significantly shorter migratory durations and earlier arrival times. While several studies have shown sex‐specific effects of candidate genes on phenology (e.g., Mettler et al., 2015; Bazzi et al., 2017), we could not test for sex effects because all 17 geolocator birds were male.

## 3 RESULTS

We successfully genotyped 72 Blackpoll Warblers at candidate genes *Clock* and *Adcyap1*. We observed four *Clock* alleles ranging in size from 186 to 195 base pairs (Table 2). We found relatively greater variation in *Adcyap1*; we detected 13 *Adcyap1* alleles ranging from 153 to 169 base pairs, five of which were found in single individuals (Table 2). Observed heterozygosity was fairly high (*H*_o_ = 0.722 for each locus), with only five individuals being homozygous at both loci. None of the populations deviated from Hardy–Weinberg equilibrium (all *p* > 0.05, Table 3), and we found no evidence of linkage disequilibrium (*p* > 0.05).

**Table 2:** Allele frequencies for two candidate genes, *Clock* and *Adcyap1,* at four populations of blackpoll warblers

Population	Clock allele lengths	Adcyap1 allele lengths															
	186	189	192	195	153	155	157	159	160	161	162	163	164	165	166	167	169
Alaska	5	12	4	7	0	1	4	13	0	4	0	4	1	1	0	0	0
Yukon	0	11	5	6	1	2	6	7	0	4	0	2	0	0	0	0	0
Manitoba	4	13	13	6	0	3	6	14	0	4	0	4	0	1	1	2	1
Eastern	6	20	15	17	0	1	10	17	1	19	1	5	2	1	0	1	0

Note

Allele numbers represent lengths in number of base pairs.

**Table 3:** Summary statistics for each candidate gene, *Clock* and *Adcyap1*, for four populations of blackpoll warbler

Population	*n*	*Clock*			*Adcyap1*			Pairwise *F*_ST_		
		*H*_o_	*H*_e_	*A*	*H*_o_	*H*_e_	*A*	Alaska	Yukon	Manitoba
Alaska	14	0.571	0.728	4	0.714	0.746	7			
Yukon	11	0.636	0.654	3	0.636	0.810	6	0.041		
Manitoba	18	0.667	0.719	4	0.722	0.806	9	−0.027	0.040	
Eastern	29	0.862	0.730	4	0.759	0.780	10	−0.001	0.049	−0.016

Note

Sample size (*n*), observed (*H*_o_) and expected heterozygosity (*H*_e_), number of alleles (*A*) per locus, and pairwise *F*_ST_. None of the *H*_o_ were statistically different from *H*_e_, and no pairwise *F*_ST_ were significant.

**Figure 3:**
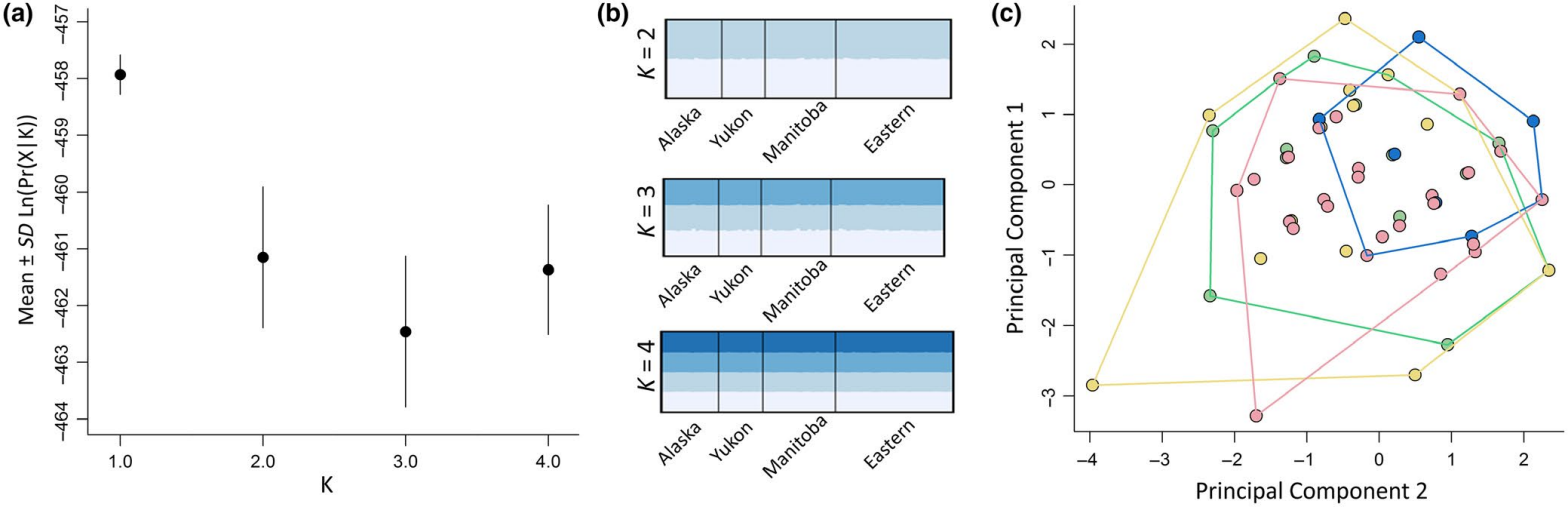
(a) Mean log probability for each number of populations (*K*) from 10 replicates in program STRUCTURE. Error bars indicate standard deviation. *K* = 1 was the most likely number of populations indicating no geographic structure. (b) Individual probability assignment to each population when *K* > 1. For all values of *K*, the assignment probability of each individual was roughly equal for all populations indicating no geographic structure. (c) Clustering of individuals according to a principal components analysis on genotype data. Colors indicate populations (Alaska = green, Yukon = blue, Manitoba = yellow, and Eastern = pink), and a minimum convex polygon was drawn around each population. All population overlap with no clustering based on population, indicating no geographic structure

We found no evidence of structured geographic variation in either *Clock* or *Adcyap1*. Overall *F*_ST_ was −0.003, and *F*_IS_ was 0.070. All pairwise *F*_ST_ values between populations were nonsignificant (Table 3), and the AMOVA revealed that only 0.72% of the variation in candidate genes was explained by difference among populations, while 99.28% corresponded to variation within populations. The STRUCTURE analysis, similarly, suggested no geographic structure with *K* = 1 as the most likely number of groups (Figure 3a). For all values of *K*, the assignment probability of each individual was roughly equal for all populations (i.e., the probability of any individual belonging to any of *K* populations ≈ 1/*K*; Figure 3b). The first two principal components from a PCA on genotype data had eigenvalues greater than 1.0 (1.61 and 1.26, respectively) and together explained 71.68% of the variation in genotype data. A PCA plot on these axes revealed no clustering by population. All populations overlapped in ordination space (Figure 3c). We found no significant relationship between allele length for either locus and either longitude or latitude, regardless of whether individual minimum, maximum, or mean allele lengths were considered (all *p* > 0.05, all *R* ^2^ values <0.05; Appendix 2). From our GLMs, we found a significant correlation between population and spring and fall departure, and spring and fall duration, but not with spring and fall arrival. These results are not explored further here, as population variation in migratory timing is more thoroughly discussed in DeLuca et al. (2019).

**Figure 4:**
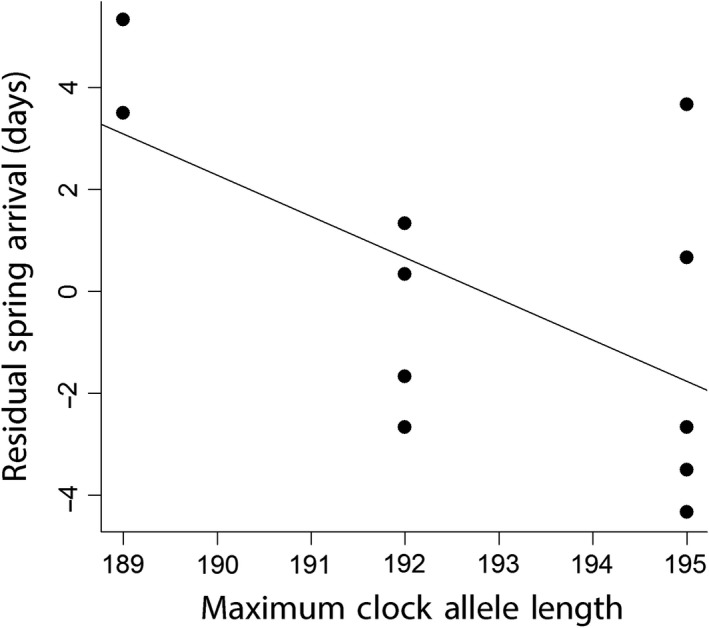
Relationship between individual maximum *Clock* allele length and spring arrival date residuals after accounting for population. Line represents slope from the general linear models with population and maximum *Clock* allele as independent variables. Individuals with longer maximum *Clock* allele lengths arrived earlier for their population compared to individuals with shorter maximum *Clock* alleles

**Figure 5:**
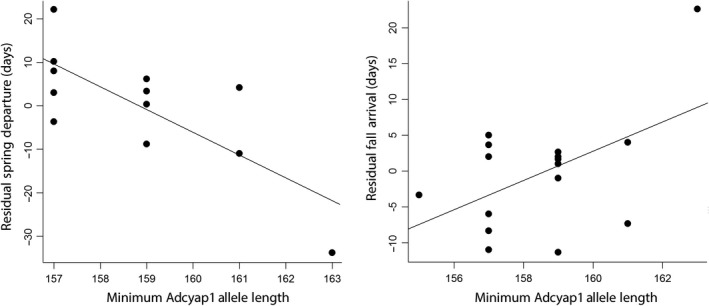
Relationship between individual minimum *Adcyap1* allele length and the residuals of spring departure and fall arrival after accounting for population. Lines represent slopes from general linear models with population and minimum *Adcyap1* allele length as independent variables. Individuals with longer minimum *Adcyap1* allele lengths departed earlier in the spring and arrived earlier in the fall for their population compared to individuals with shorter minimum *Adcyap1* alleles

Despite finding no evidence of geographic variation in candidate genes, we did find evidence that both *Clock* and *Adcyap1* allele length were correlated with migratory behaviors at an individual level (Table 4). Maximum *Clock* allele length was significantly and negatively associated with spring arrival date on the breeding ground (GLM: *F*_3,7_ = 9.466, *p* = 0.007, *R* ^2^ = 0.718; *β*_MaxClock_ = −1.25, *p*_MaxClock_ = 0.024); individuals with longer Clock alleles tended to arrive to the breeding territories earlier in the spring (Figure 4). We found no other correlations for *Clock* allele length and the other five migration traits from our single gene models (Appendix 3). We found significant correlation between individual's minimum *Adcyap1* allele length and the timing of spring and fall migration. Blackpoll warblers with longer minimum *Adcyap1* alleles departed earlier in the spring (*F*_3,8_ = 6.936, *p* = 0.013, *R* ^2^ = 0.618; *β*_MinAdcyap1_ = −5.615, *p*_MinAdcyap1_ = 0.007) and arrived later to the southern wintering grounds (*F*_3,13_ = 2.418, *p* = 0.113, *R* ^2^ = 0.210; *β*_MinAdcyap1_ = 2.122, *p*_MinAdcyap1_ = 0.043; Figure 5). A single individual that was homozygous for the longest minimum *Adcyap1* allele had by far the earliest spring departure, the longest spring and fall duration, and the latest fall arrival. Removal of this individual from GLM analyses resulted in nonsignificant relationships between minimum *Adcyap1* allele length and both spring departure (*F*_3,7_ = 8.531, *p* = 0.010, *R* ^2^ = 0.693; *β*_MinAdcyap1_ = −3.239, *p*_MinAdcyap1_ = 0.088) and fall arrival (*F*_3,12_ = 2.469, *p* = 0.112, *R* ^2^ = 0.227; *β*_MinAdcyap1_ = 0.580, *p*_MinAdcyap1_ = 0.535). The correlation between *Adcyap1* and fall arrival was also significant when considering individuals' mean allele length (Table 4). Results were consistent when using longitude and latitude as covariates instead of population.

**Table 4:** General linear model results for individual‐level analyses of blackpoll warbler candidate gene alleles and migratory traits

Gene	Migratory trait	Allele	*n*	*β*_Allele Length_	*SE*	*p*_Allele Length_	*R* ^2^_Allele Length_
*Clock*	Spring arrival	Max	11	−1.250	0.438	0.024	0.538
*Adcyap1*	Spring departure	Min	12	−5.615	1.577	0.007	0.618
	Spring duration	Min	12	5.123	1.657	0.015*	0.544
	Fall duration	Min	17	1.941	0.588	0.006*	0.456
		Mean	17	1.844	0.797	0.038*	0.292
	Fall arrival	Min	17	2.122	0.945	0.043	0.279
		Mean	17	2.889	1.053	0.017	0.367

Note

Allele indicates whether an individual's minimum, maximum, or mean allele length was used for each analysis. Slope (*β*), *p*‐value, and partial *R* ^2^ values are given for allele length and each migratory trait. Population was also used as a predictor variable, though the slopes and *p*‐values for this factor are not provided. Only models for which allele length was significant (*p* ≤ 0.05) are shown. Results for all general linear models provided in Appendix 3.

* Significant single gene effects that are not further discussed because of a significant genetic interaction effect on this migratory trait (see Table 5).

**Figure 6:**
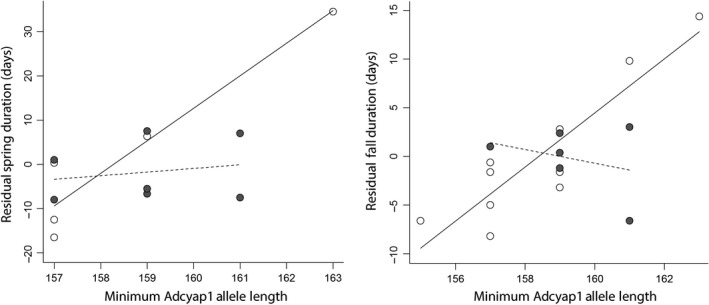
GLM results for an interaction effect between mean *Clock* and minimum *Adcyap1* allele lengths on spring and fall migratory duration. White circles and solid lines represent individuals with shorter‐than‐average mean *Clock* allele lengths, and gray circles and dashed lines represent individuals with longer‐than‐average mean *Clock* allele lengths. Gene interactions were significantly correlated (*p* < 0.05) with both spring and fall duration. Migratory duration shows a significant positive relationship with minimum *Adcyap1* allele length in both seasons when *Clock* alleles are short, but not when *Clock* alleles are long

We also found an interaction effect between *Clock* and *Adcyap1* allele lengths on migratory duration (Figure 6). When we used mean *Clock* allele length and minimum *Adcyap1* allele length, this interaction was significantly correlated with both spring duration (*F*_5,6_ = 10.15, *p* = 0.007, *R* ^2^ = 0.806; *p*_MeanClock_ = 0.020, *p*_MinAdcyap1_ = 0.018, *p*_MeanClock × MinAdcyap1_ = 0.019) and fall duration (*F*_5,11_ = 22.38, *p* < 0.001, *R* ^2^ = 0.870; *p*_MeanClock_ = 0.007, *p*_MinAdcyap1_ = 0.007, *p*_MeanClock × MinAdcyap1_ = 0.007). For individuals with shorter‐than‐average mean *Clock* allele lengths, both spring duration and fall duration were determined by a significant positive relationship with minimum *Adcyap1* allele length. For individuals with longer‐than‐average mean *Clock* allele lengths, duration was not correlated with *Adcyap1* length (Figure 6). When the individual with the longest minimum *Adcyap1* allele is removed from the analyses, this relationship becomes nonsignificant for spring duration (*F*_5,5_ = 5.538, *p* = 0.042, *R* ^2^ = 0.694; p_MeanClock × MinAdcyap1_ = 0.120), but remains significant for fall duration (*F*_5,10_ = 23.37, *p* < 0.001, *R* ^2^ = 0.882; *p*_MeanClock × MinAdcyap1_ = 0.011).This *Clock* × *Adcyap1* interaction effect was weaker when using either maximum *Clock* allele length or mean *Adcyap1* allele length (Table 5).

**Table 5:** General linear model results for interaction effects between candidate genes on spring and fall duration

*Clock* allele	*Adcyap1* allele	Migratory trait	*β_Clock_*_ × _ *_Adcyap1_*	*p_Clock_*_ × _ *_Adcyap1_*
Mean	Min	**Spring duration**	**−1.910**	**0.019**
		**Fall duration**	**−0.740**	**0.007**
	Mean	Spring duration	−0.143	0.934
		**Fall duration**	**−0.720**	**0.029**
Max	Min	Spring duration	−1.450	0.056
		**Fall duration**	**−0.605**	**0.042**
	Mean	Spring duration	0.658	0.671
		Fall duration	−0.558	0.08

Note

*Clock* and *Adcyap1 a*llele columns indicate whether an individual's minimum, maximum, or mean allele length was used for each analysis. Slope (*β*) and *p*‐value are given for the interaction term. Population was also used as a predictor variable, though the slope and *p*‐values for this factor are not provided. GLMs were only run using mean or max *Clock* allele lengths and minimum or mean *Adcyap1* allele lengths based on results from single gene models. Only models with spring or fall duration as the dependent variable are presented here; no other migratory traits had a significant interaction term. Models with significant interaction effects (*p* ≤ 0.05) are bolded.

Lastly, we tested for differences in each of the migratory traits between homozygotes and heterozygotes of each locus. We found individuals that were heterozygous at *Adcyap1* had significantly shorter spring duration after accounting for population origin in a GLM (mean homozygote residuals = 8.47; mean heterozygote residuals = −8.47, *p* = 0.030) and significantly shorter fall duration (mean homozygote residuals = 3.26; mean heterozygote residuals = −2.90, *p* = 0.031) compared to homozygotes.

## 4 DISCUSSION

We found evidence suggesting allele lengths in candidate genes *Clock* and *Adcyap1* are correlated with spring and fall migratory behavior in blackpoll warblers. Despite no geographic variation across populations in allele frequencies or lengths, variation in candidate gene allele lengths was associated with nearly every migratory trait analyzed at the individual level. It is worth noting that we conducted a large number of statistical tests (*N* = 78 GLMs), increasing the chance of type I errors. However, with *α* = 0.05, we would we expect an average of 3.7 of the 78 tests to yield significant results at random. We find significant correlations (*p* < 0.05) in 12 cases, significantly more than expected at random (binomial test, *p* < 0.001). Given that the observed relationships are mostly in agreement with predicted direction of relationships, and are consistent with studies in other species, it is likely that our results represent real relationships between the variables and not spurious statistical effects. While sample sizes are low given the low recovery rates of geolocator‐fitted birds (DeLuca et al., 2019), future work that aims to increase sample sizes may allow more rigorous evaluation of links between candidate genes and migration behavior. For example, with more birds, analyses could control for other factors known to influence migratory timing and speed, such as age, sex, or environmental conditions experienced during and leading up to migration, which could obscure genetic contributions to migration phenology.

The only consistent effect we found for *Clock* alleles was a negative correlation with spring arrival date (Figure 4, Table 4). This was a surprising result as all other published studies that report a significant effect of *Clock* allele length on spring phenology in birds report a positive correlation (Table 1; Bazzi et al., 2015; Bourret & Garant, 2015; Caprioli et al., 2012; Liedvogel et al., 2009; Saino et al., 2015). Why blackpoll warblers would exhibit an opposite pattern at this locus is unclear, though this highlights the emerging pattern of variation across species in migratory genotype–phenotype relationships and a need for further detailed studies that investigate the functional influence of candidate gene alleles. Previous studies have suggested that longer *Clock* alleles are dominant over shorter alleles in their influence on phenology (Bazzi et al., 2015; Saino et al., 2015). Our results seem to support this given that we find a stronger relationship between maximum *Clock* allele length and spring arrival time, compared to minimum *Clock* alleles.

Our results showed longer minimum *Adcyap1* alleles were associated with earlier spring departure and later fall arrival on the wintering grounds. These results are generally consistent with work previously published on other species (Table 1). While no other studies to our knowledge have shown a significant correlation between *Adcyap1* and arrival date on the wintering grounds as we show here, these results are consistent with an effect of *Adcyap1* on longer fall migration duration (Mueller et al., 2011; Peterson et al., 2013). The strongest relationships between migratory traits and *Adcyap1* were with an individual's minimum allele length (Appendix 3). This may suggest dominance of the shortest *Adcyap1* allele, a finding not reported in other species. However, the one individual in our sample that was homozygous for the longest *Adcyap1* allele (163 bp) had by far the earliest spring departure, the longest spring and fall duration, and the latest fall arrival. This may support that the effects of long *Adcyap1* alleles on migratory behavior are additive instead of dominant, as was previously suggested by Bourret and Garant (2015). Future studies with larger sample sizes may be able to more directly assess dominance by comparing migratory traits across genotypes at the individual level (Liedvogel et al., 2009; Saino et al., 2015). Our results that a single individual with a rare genotype differs greatly in migratory behavior are also consistent with studies published on other species that show a few individuals with rare genotypes can show significantly different migratory traits compared to the rest of the population (Bazzi et al., 2015). For example, a single individual barn swallow with the longest observed *Clock* allele had significantly later migration in both spring and fall compared to the rest of the population (Bazzi et al., 2015).

We found evidence of a significant interaction between *Clock* and *Adcyap1* allele lengths on migratory duration, the first such finding in studies of migratory birds to our knowledge. Previous studies that have investigated the effects of both *Clock* and *Adcyap1* either did not find a significant interaction between genes or did not test for one (Bazzi et al., 2016, 2017; Bourret & Garant, 2015; Contina et al., 2018; Peterson et al., 2013; Saino et al., 2015). In this interaction in our study, *Adcyap1* length appears to increase migration duration when corresponding *Clock* allele length is short, especially in the fall. The peptide product of *Adcyap1*, PACAP, regulates the expression of *Clock* in chicken (*Gallus gallus domesticus*) pineal glands (Nagy & Csernus, 2007), which may suggest the interaction effect we observe is the result of differential expression of *Clock* as determined by *Adcyap1* allele length. However, it is yet unknown how *Adcyap1* allele length influences PACAP structure or function, including downstream regulation of *Clock*. These complex genetic interactions may explain in some cases why previous studies have failed to identify effects in single candidate genes.

Our findings that candidate gene allele lengths are correlated with five of the six migratory traits raise an important question about whether the migratory phenotypic characters assessed in this study and others are evolutionarily independent of one another. For example, is variation in arrival dates a secondary consequence of natural selection on departure date and migratory duration, or might selection be acting on these characters separately and independently? Among the 17 blackpoll warblers used in this study, duration and departure date for both spring and fall migration were strongly and negatively correlated (spring *r* = −0.948, fall *r* = −0.677), suggesting that birds who departed later had a shorter duration (faster rate of migration). However, duration and arrival date were not correlated or only moderately so for spring and fall, respectively (spring *r* = −0.010, fall *r* = 0.457). While a study with similar sample sizes in another species showed a close relationship between departure and arrival dates (Ouwehand & Both, 2017), surprisingly, departure and arrival dates were weakly correlated in our data for both seasons (spring *r* = 0.270, fall *r* = 0.290). These data suggest migratory departure and arrival dates may be independent of one another. Further, variation in duration may be more closely tied to variation in departure date than arrival date, perhaps due to stronger selection on arrival date especially in the spring (Nilsson et al., 2013). Our data also showed a strong positive correlation between spring and fall duration (*r* = 0.688), and both of these characters were associated with a significant interaction between *Clock* and *Adcyap1* (Figure 6). Whether these genes influence spring and fall duration via a common mechanism, perhaps through increased migratory restlessness (Mueller et al., 2011; Peterson et al., 2013), or whether these characters are independent requires further investigation. Nilsson et al. (2013) examined timing and speed of fall and spring migration in published tracking studies and found evidence for stronger selection on spring migratory phenology compared to fall, potentially suggesting evolutionary independence of these phenotypic characters.

Part of the answer to this question about evolutionary independence depends on the control of these characters at a proximate molecular level. Are there separate molecular pathways that control departure dates and duration, or that control spring and fall duration, or are these all proximately linked? PACAP is known to have broad influence on the physiology and behavior of organisms, acting in the brain and throughout peripheral organs (Mueller et al., 2011; Vaudry et al., 2009). It is therefore plausible that this gene could influence similar behaviors in separate seasons via independent molecular pathways. Conversely, it is perhaps equally plausible that a common molecular pathway is triggered by environmental cues in multiple seasons. This again highlights the need for studies that further investigate the functional role of candidate genes, how they influence migratory behaviors throughout the annual cycle, both independently and interactively with other factors (e.g., age, sex, environmental conditions), and specifically how variation in allele length influences the expression level, structure, and molecule functioning of gene products.

Although we found relationships between candidate genes and migratory traits at the individual level, we found no geographic structure in candidate genes. One possible explanation for this is that geographic variation in migratory behavior is explained by variation in environmental cues and not local adaptation in candidate genes. Geographic variation in behavior may be the result of plastic responses to variable environments, independent of the effects of those environments on selection in candidate genes (Foster, 1999). Therefore, to the extent environmental cues vary across breeding (or wintering) sites, it is possible we might observe behavioral differences in migratory behaviors without underlying geographic differences in genes. For example, during fall migration western breeding blackpoll warblers depart earlier and take longer to arrive at wintering grounds compared to eastern breeding birds (DeLuca et al., 2015, 2019), despite no differences in candidate gene frequencies between those populations. Our results suggest these differences across populations could be the results of plastic responses to differing environmental cues, while candidate genes are still correlated with variation in timing among individuals within each population. Further, Bourret and Garant (2015) point out that gene‐by‐environment interactions are underappreciated in the study of candidate genes. In their study of breeding phenology in tree swallows (*Tachycineta bicolor*), they found most candidate gene genotype–phenotype relationships were affected by environmental variables such as breeding density, latitude (a proxy of photoperiod), and temperature (Bourret & Garant, 2015). If genotype–phenotype relationships are influenced by environment, again we may observe behavioral differences across populations without underlying population genetic differences. Alternatively, it might simply be that any interpopulation variation in allele frequencies or lengths are undetectable given high levels of intrapopulation variation and our relatively small sample size; however, our result of no geographic structure of candidate genes is consistent with studies in other bird species (Bazzi et al., 2016; Dor et al., 2012, 2011; Johnsen et al., 2007). It is unlikely that the lack of geographic structure in candidate genes in blackpoll warblers is due solely to admixture from gene flow between locally adapted populations. Ralston and Kirchman (2012) found genetic structure due to isolation by distance at the continental scale in this species, despite a relatively high number of shared alleles among populations (Ralston & Kirchman, 2012, 2013). Future studies may benefit from comparing geographic patterns in candidate and neutral loci (Contina et al., 2018).

We observed greater allelic diversity in blackpoll warblers at *Adcyap1* than at *Clock*, a pattern that appears to be typical across birds (Bazzi et al., 2016; Contina et al., 2018; Peterson et al., 2013; Steinmeyer et al., 2009). However, our results differ in that we find relatively high heterozygosity (74.3%) at the *Clock* locus, despite only observing four alleles. In studies of other bird species, *Clock* heterozygosity was quite low (0%–9%; Bazzi et al., 2015, 2016; Chakarov et al., 2013; Dor et al., 2011). Low heterozygosity at microsatellite loci may be the result of stabilizing selection or loss of diversity due to inbreeding and therefore useful in studies of individual fitness (Chapman, Nakagawa, Coltman, Slate, & Sheldon, 2009; Dor et al., 2011). While a single microsatellite locus is not sufficient in estimating genome‐wide heterozygosity or inbreeding (Miller et al., 2014), individual heterozygosity at a small number of microsatellite loci can be useful in the study of avian life history traits and fitness (Forstmeier, Schielzeth, Mueller, Ellegren, & Kempenaers, 2012; Lens et al., 2000), especially if those loci are functionally important and under selection (Chapman et al., 2009). *Adcyap1* heterozygosity in Eurasian blackcaps was significantly associated with earlier spring arrival (Mettler et al., 2015), perhaps suggesting these individuals were of greater migratory fitness and able to migrate more quickly. We found similar results in blackpoll warblers in that heterozygotes at *Adcyap1* had shorter spring and fall duration than homozygotes. We found no associations between heterozygosity at *Clock* and any migratory trait. Few studies of migratory candidate genes have examined the effect of individual heterozygosity, but our results and those of Mettler et al. (2015) suggest this is a potentially fruitful avenue of further investigation.

The results of our individual‐level analyses demonstrate the value of studies that reveal individual migratory phenotypes, for example, by using light‐level geolocators (McKinnon & Love, 2018). The combination of geolocator data with candidate gene analysis has been an important advancement in the study of migration (Bazzi et al., 2015; Contina et al., 2018; Saino et al., 2017). For example, in the study of barn swallows, individual‐level analyses revealed that rare *Clock* genotypes can have a significant impact on migratory phenology (Bazzi et al., 2015) and that degree of DNA methylation at the *Clock* gene can explain individual variation in migratory behavior (Saino et al., 2017). Both of these insights would likely have been missed with analysis of population‐level genetic and migratory variation. Increasing the number of species and individuals with linked genotype–phenotype information will allow richer investigations of migratory candidate genes.

Understanding variation in candidate genes across species is especially important for behaviors that are evolutionarily labile and may have arisen independently in multiple lineages. We therefore encourage future studies of candidate migratory genes to investigate species that are codistributed, share a biogeographic history, or are in the same family as those species that have already been studied. This will allow a better understanding of the influences of environment and history on selection at candidate genes, as well as the degree to which these patterns are conserved within lineages.

## CONFLICT OF INTEREST

None declared.

## AUTHOR CONTRIBUTIONS

JR, LL, and MM conceived of the project, performed the genetics laboratory work and statistical analyses, and wrote the paper. All other authors contributed to field efforts to collect blood samples or retrieve geolocators, and provided critical feedback on analyses and the manuscript. WVD took the lead on geolocator data analysis.

## Data Availability

All data used in analyses in the paper, including *Clock* and *Adcyap1* genotype information, capture coordinates, and migratory trait values are available in Dryad Digital Repository (https://doi.org/10.5061/dryad.d10qb58). An R script is available in supplementary materials (Data S1) and can be used in combination with the dataset on Dryad to perform all analyses in this paper.

## APPENDIX 1

Tissue and blood sampling locations, population assignments for the current study, and original tissue sources (population, sample location, source, *n*)

**Table:**

Population (*n*)	Source	Source *n*	Catalog numbers	Geolocator numbers
Alaska (14)	Current study (blood)^a^	10		3254‐001, 3254‐003, 3254‐008, 3254‐011, 3254‐05
	University of Alaska Museum (frozen tissue)	3	UAM7394, UAM20089, UAM20508	
	UCLA Conservation Genetics Resource Center (feather)	1	01N9262	
Yukon (11)	Current study (blood)^a^	11		blpw12, blpw14, blpw15, blpw25
Manitoba (18)	Current study (blood)^a^	17		4105‐002, 4105‐004, 4105‐006, 4105‐008, 4105‐009, 4105‐010, 4105‐016, 4105‐017
	Ralston and Kirchman (2012; blood)^b^	1		
Eastern (29)	Current study (blood)^a^	10		
	New York State Museum (frozen tissue)	4	NYSM11077, NYSM11078, NYSM11079, NYSM11080	
	Ralston and Kirchman (2012; blood)^b^	15		

a Blood samples and DNA extracts archived at Saint Mary's College, Notre Dame, Indiana, USA.

b Blood samples and DNA extracts archived at New York State Museum, Albany, New York, USA.

## APPENDIX 2

General linear model results for variation in allele lengths by longitude and latitude. There was no significant relationship between allele length for either locus and either longitude or latitude, regardless of whether individual minimum, maximum, or mean allele lengths were considered

**Table:**

Gene	Allele	*β*_Longitude_	*p*_Longitude_	*β*_Latitude_	*p*_Latitude_	*F*	*df*	*p*
*Clock*	Min	0.005	0.720	0.004	0.544	0.220	2,69	0.804
*Clock*	Mean	0.008	0.466	0.018	0.759	0.417	2,69	0.664
*Clock*	Max	0.011	0.388	−0.006	0.926	1.437	2,69	0.245
*Adcyap1*	Min	−0.004	0.776	−0.055	0.417	0.579	2,69	0.563
*Adcyap1*	Mean	0.007	0.555	0.003	0.957	0.483	2,69	0.619
*Adcyap1*	Max	0.017	0.209	0.062	0.389	0.859	2,69	0.428

## APPENDIX 3

General linear model results for individual‐level analyses of blackpoll warbler candidate gene alleles and migratory traits. Allele indicates whether an individual's minimum, maximum, or mean allele length was used for each analysis. Slope (*β*) with standard error (*SE*), *p*‐value, and partial *R* ^2^ are given for the relationship between allele length and each migratory trait. Population was also used as a predictor variable, though the slope and *p*‐values for this factor are not provided. Models with a significant slope for allele length (*p* ≤ 0.05) are shown with bold text.

**Table:**

Gene	Migratory trait	Allele	*n*	*β*_AlleleLength_	*SE*	*p*_AlleleLength_	*R* ^2^_AlleleLength_
*Clock*	Spring departure	Min	12	−0.258	1.811	0.890	0.003
		Mean	12	−0.488	2.156	0.827	0.006
		Max	12	−0.810	2.478	0.752	0.013
	Spring duration	Min	12	−0.333	1.752	0.854	0.005
		Mean	12	−0.353	2.09	0.870	0.004
		Max	12	−0.310	2.413	0.901	0.002
	Spring arrival	Min	11	−0.626	0.384	0.147	0.275
		Mean	11	−0.912	0.425	0.069	0.397
		Max	11	**−1.250**	**0.438**	**0.024**	**0.538**
	Fall departure	Min	17	−0.102	0.543	0.854	0.003
		Mean	17	−0.533	0.633	0.415	0.052
		Max	17	−0.923	0.599	0.147	0.154
	Fall duration	Min	17	−0.369	0.577	0.534	0.03
		Mean	17	0.065	0.702	0.927	0.001
		Max	17	0.662	0.679	0.347	0.068
	Fall arrival	Min	17	−0.556	0.804	0.502	0.035
		Mean	17	−0.574	0.967	0.563	0.026
		Max	17	−0.354	0.977	0.723	0.01
*Adcyap1*	Spring departure	Min	12	**−5.615**	**1.577**	**0.007**	**0.613**
		Mean	12	−2.646	2.575	0.334	0.117
		Max	12	0.611	1.893	0.755	0.013
	Spring duration	Min	12	**5.123**	**1.657**	**0.015**	**0.544**
		Mean	12	2.435	2.509	0.360	0.105
		Max	12	−0.537	1.835	0.777	0.011
	Spring arrival	Min	11	−0.742	0.543	0.214	0.211
		Mean	11	−0.322	0.645	0.633	0.034
		Max	11	0.106	0.462	0.826	0.007
	Fall departure	Min	17	0.122	0.738	0.871	0.002
		Mean	17	0.522	0.866	0.557	0.027
		Max	17	0.470	0.631	0.470	0.041
	Fall duration	Min	17	**1.941**	**0.588**	**0.006**	**0.456**
		Mean	17	**1.844**	**0.797**	**0.038**	**0.292**
		Max	17	0.512	0.681	0.465	0.042
	Fall arrival	Min	17	**2.122**	**0.945**	**0.043**	**0.279**
		Mean	17	**2.889**	**1.053**	**0.017**	**0.367**
		Max	17	1.500	0.878	0.111	0.183
